# Differentiation of hypovascular pancreatic neuroendocrine tumors from pancreatic ductal adenocarcinoma using contrast-enhanced computed tomography

**DOI:** 10.1371/journal.pone.0211566

**Published:** 2019-02-01

**Authors:** Shuai Ren, Xiao Chen, Zhonglan Wang, Rui Zhao, Jianhua Wang, Wenjing Cui, Zhongqiu Wang

**Affiliations:** Department of Radiology, the Affiliated Hospital of Nanjing University of Chinese Medicine, Nanjing, China; UKSH Campus Lübeck, GERMANY

## Abstract

Hypovascular pancreatic neuroendocrine tumors (hypo-PNETs) are often misdiagnosed as pancreatic ductal adenocarcinoma (PDAC). However, the treatment options and prognosis of PNETs and PDAC are substantially different. This retrospective study differentiated hypo-PNETs from PDAC using contrast-enhanced CT (CE-CT). Clinical data and CE-CT findings, including tumor location, size, boundary, pancreatic duct dilatation, local invasion or metastases, tumor contrast enhancement, and tumor-to-pancreas enhancement ratio, were compared between 39 PDACs and 18 hypo-PNETs. At CT imaging, hypo-PNETs showed a higher frequency of a well-defined margin and lower frequencies of pancreatic duct dilatation and local invasion or metastasis when compared with PDAC (*p < 0*.*05* for all). The mean attenuation of hypo-PNETs at the arterial and portal venous phase was significantly higher than that of PDAC (*p* < 0.001, *p* = 0.003, respectively). Similar results were observed in tumor-to-pancreas enhancement ratio. Tumor attenuation and tumor-to-pancreas enhancement ratio at the arterial phase showed the largest area under the curve (AUC) of 0.888 and 0.812 with 83.3–88.9% of sensitivity and 61.6–77.0% of specificity. Pancreatic duct dilatation, local invasion or metastasis, and tumor attenuation at the portal venous phase also showed acceptable AUC (0.703–0.748). Thus CE-CT features, especially the enhancement degree at the arterial phases, may be useful for differentiating hypo-PNETs from PDAC using CE-CT.

## 1. Introduction

Pancreatic neuroendocrine tumors (PNETs) constitute a heterogeneous group of tumors that originate from neuroendocrine cells [1]. PNETs are regarded as potentially malignant tumors [2]. The incidence of PNETs is as high as 10% in pancreatic tumors [1]. The current detection rate of incidental PNETs during CT or MRI imaging procedures is increasing [3, 4].

According to the World Health Organization (WHO) classification, PNETs were categorized into low grade (G1), intermediate grade (G2), and high grade (G3) based on the Ki-67 index and the mitotic activity [5]. However, G3 PNET is more heterogeneous than expected and the survival outcome differs among PNET G3 patients. Therefore, WHO classification was revised in 2017 [6, 7]. Previous G3 neuroendocrine carcinoma was divided into well-differentiated neuroendocrine tumor (NET G3) and poorly-differentiated neuroendocrine neoplasm (NEC G3), and the latter was further divided into small cell type and large cell type [8].

Contrast-enhanced CT (CE-CT) is the primary imaging modality for evaluating the pancreas [1]. Previous reports have shown that PNETs are well-defined masses with hypervascular enhancement patterns, absent of ductal dilatation, and lack of vascular encasement [9, 10]. However, some recent studies have demonstrated that G2 or G3 PNETs can also show ill-defined margins, hypovascular enhancement, and pancreatic duct dilatation [3, 11, 12], which are typical imaging features of pancreatic ductal adenocarcinoma (PDAC). Notably, the treatment options and prognosis of PNETs and PDAC are substantially different, PNETs reveal a higher resectability and have a better response to chemotherapy [10, 13]. Surgery remains the treatment of choice for any localized PNET, as is associated with a significantly higher 5-year survival [3, 14], which is higher than that of PDAC (< 5%). In addition, somatostatin analogues or ablation may benefit patients with unresectable or residual disease [15, 16]. Considering the repeated biopsy is invasive and difficult, there is great clinical value in better differentiating hypovascular PNETs (hypo-PNETs) from PDAC with preoperative imaging.

Several previous reports have shown the differentiation of PNEC or nonhypervascular PNETs from PDAC on MRI imaging [14, 17]. To the best of our knowledge, only few reports demonstrated the values of CE-CT features in differentiating hypo-PNETs from PDAC. Kim et al. [1] showed the differential diagnosis of PDAC from PNETs. However, hypervascular PNETs were also included in their study. In the present study, we showed the values of CT features in differentiating hypo-PNETs from PDAC.

## 2. Materials and methods

### Patient selection

**Fig 1:**
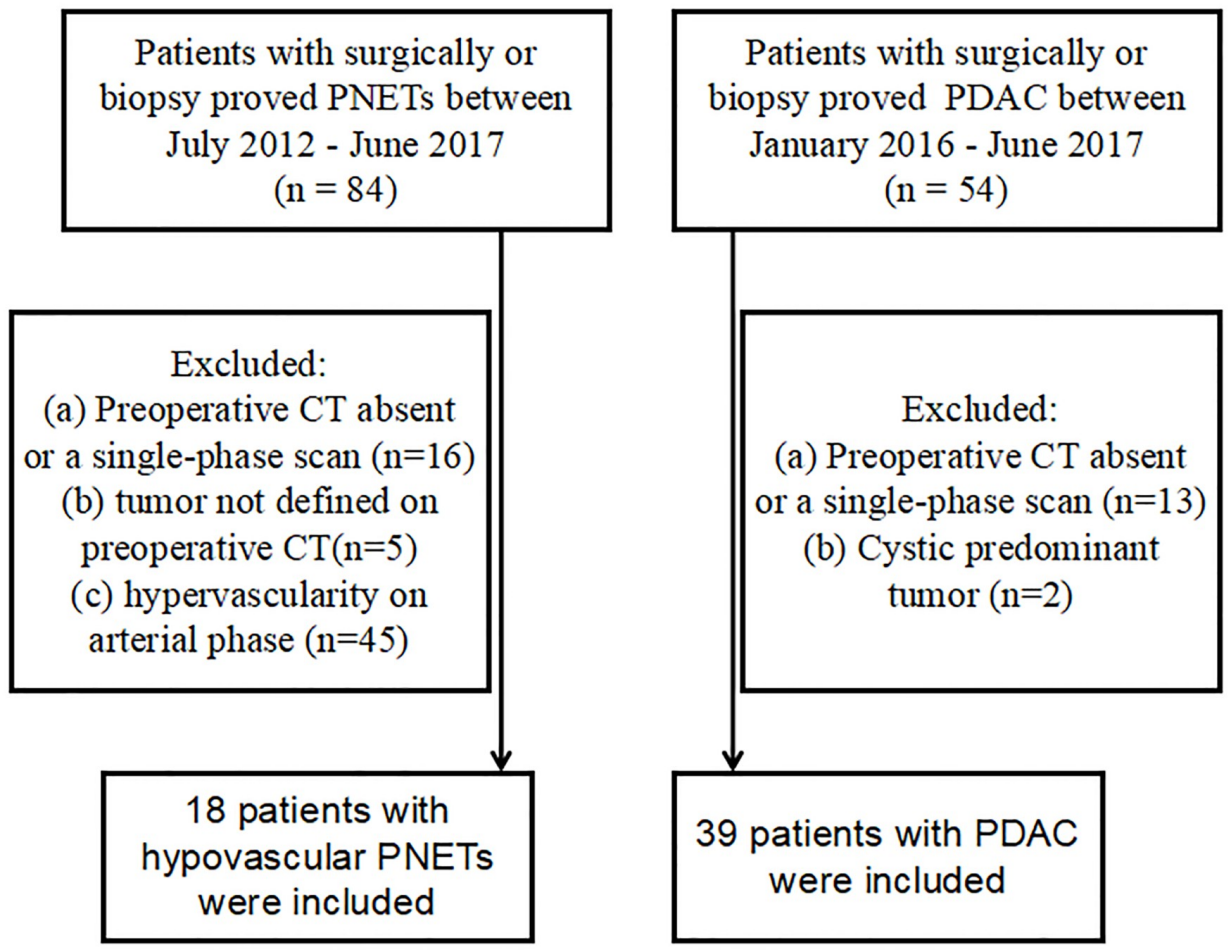
Flow diagram of patient inclusion. PNET, pancreatic neuroendocrine tumor; PDAC, pancreatic ductal adenocarcinoma.

This study was approved by the institutional review board of the Affiliated Hospital of Nanjing University of Chinese Medicine and patient informed consent was waived due to its retrospective nature. Eighty-four patients with pathologically confirmed PNETs through needle biopsy or postoperative specimen between July 2012 and June 2017 were found in our medical database. Due to the incidence of PDAC being higher than that of PNETs, especially hypo-PNETs, the inclusion period of time for hypo-PNETs was longer than that of PDAC. Those masses which showed lower enhancement levels than those of the adjacent pancreatic parenchyma at the arterial phase were considered as hypo-PNETs. Exclusion criteria were as follows: (a) Preoperative CT absent or a single-phase scan (n = 16); or (b) tumor not defined on preoperative CT (n = 5); or (c) tumor presented with a hypervascular pattern on CE-CT images (n = 45). Finally, 18 patients (8 men and 10 women) with hypo-PNETs were included in our study (Fig 1). The mean age was 59.2 ± 10.1 years (age range, 43–76).

Similarly, 54 patients with pathologically confirmed PDAC through needle biopsy or postoperative specimen between January 2016 and June 2017 were found in our medical database. Exclusion criteria were as follows: (a) Preoperative CT absent or a single-phase scan (n = 13); or (b) tumor presented as dominantly cystic (n = 2). Finally, 39 patients (26 men and 13 women) with PDAC were included in our study (Fig 1). The mean age was 64.1 ± 8.8 years (age range, 39–78).

### CT imaging technique

CT images were obtained by different multi-detector CT (MDCT) scanners. The following CT machines were used: Philips Brilliance 64 (Philips Healthcare, DA Best, the Netherlands), Discovery HD750 (GE Healthcare, Milwaukee, Wisconsin, USA), and optima 670 (GE healthcare, Tokyo, Japan). Dynamic CT images consisting of the unenhanced, arterial, portal venous and delayed phase images were obtained. All CE-CT were with the intravenous administration of Ultravist (Ultravist 300, Bayer Schering Pharma AG, 1.2 ml/kg body weight) at a rate of 3.0 ml/s followed by 40ml saline solution through the elbow vein via a power injector. The imaging parameters were as follows: tube voltage, 120 kVp; tube current, 200–400 mAs; a helical pitch of 1.375; slice thickness, 3.0 mm; slice interval, 3.0 mm, and a reconstruction interval of 1.25 mm. Fourteen cases of PNETs and 31 cases of PDAC patients underwent a 4-phase CT examination (unenhanced, arterial, portal venous, and delayed phase). The other 4 cases of PNETs and 8 cases of PDAC patients underwent a 3-phase CT examination (unenhanced, arterial and portal venous phase). The mean imaging time delay was 30 s for the arterial phase, 60s for the portal venous phase, and 120s for the delayed phase.

### CT image analysis

Two radiologists (S.R. and R. Z., with 5 and 6 years of experience in abdominal radiology, respectively) with no prior knowledge of detailed histopathological information of any patients, reviewed CT images independently. If there was inconformity, consensus was reached through discussion or referral to a third radiologist (J.H.W., with 11 years of experience in abdominal radiology). The following CT imaging findings were evaluated: tumor location (head, body, or tail), tumor margin (well or ill-defined margin), calcification, pancreatic duct dilatation, common bile duct dilatation, pancreatic atrophy, and local invasion or metastases. A well-defined margin was defined as a smooth and clearly visible margin. A poorly-defined margin was defined as spiculation or infiltration on > 90% of tumor perimeter [15]. The presence of calcification was defined on the unenhanced phase images. Pancreatic duct dilatation was defined as the main duct diameter ≥ 4mm. Bile duct dilatation was confirmed as both the extrahepatic bile duct (> 8mm) and the intrahepatic bile duct (> 2mm) [12].

The size (cm) and CT attenuation (HU) of the tumors and the adjacent parenchyma were measured at each phase by another radiologist (W.J.C., with 15 years of experience in abdominal radiology). CT attenuation of the tumors and the adjacent parenchyma was determined by drawing a region of interest (ROI) in the solid part of tumor showing the most remarkable enhancement and the downstream parenchyma as large as possible. Intratumoral calcification and cystic or necrotic components were avoided during the measurements of CT attenuation. Then the ROI was placed on the same equivalent site for each phase CT. The tumor-to-pancreas enhancement ratio was defined as the attenuation (HU) of pancreatic lesion divided by that of the pancreatic parenchyma measured at each phase of MDCT. For all images, each ROI measured 3 times and the average value was calculated.

### Statistical analysis

Categorical variables were presented as the number of cases (percentage) and were analyzed by using Chi-square or Fisher’s exact tests and quantitative variables were presented as mean ± SD, and were analyzed by Mann-Whitney U test. The receiver operating characteristic (ROC) curves were adopted and cut-off values were calculated to determine the performance of CT features in differentiating hypo-PNETs from PDAC. A p-value < 0.05 was considered a significantly statistical difference. All statistical analyses were performed by using SPSS (Version 20.0 IBM Corp. IBM SPSS Statistics for Windows).

## 3. Result

### Patient and tumor characteristics

Characteristics of the 18 hypo-PNETs and 39 PDAC patients are summarized in Table 1. Eighteen patients with pathologically confirmed PNETs through needle biopsy (3 cases) or surgery (15 cases) and 39 patients with pathologically confirmed PDAC through needle biopsy (5 cases) or surgery (34 cases) were included and compared in our study. No significant differences were found in age, gender, or clinical symptoms between the two groups. However, yellow urine or icterus was more common in PDAC compared with hypo-PNETs patients (33.3% vs. 11.1%, p > 0.05). PDAC tended to occur in men compared with hypo-PNETs (66.7% vs. 44.4%, p > 0.05).

**Table 1:** Demographic characteristics of patients with hypovascular pancreatic neuroendocrine tumors (hypo-PNETs) and those with pancreatic ductal adenocarcinoma.

Variables	hypo-PNETs (n = 18)	PDAC (n = 39)	*P* value
Age (years)	59.2±10.1 (43–76)	64.1±8.8 (39–78)	0.07^a^
Gender			0.112^b^
Male	8 (44.4)	26 (66.7)	
Female	10 (55.6)	13 (33.3)	
Clinical symptoms			
Abdominal pain	14(77.8)	27(69.2)	>0.05^b^
Abdominal bloating or diarrhea	3(16.7)	9(23.1)	>0.05^b^
Yellow urine or icterus	2(11.1)	13(33.3)	>0.05^b^
Marasmus	1(5.56)	5(12.8)	>0.05^b^
Asymptomatic	4(22.2)	8(20.5)	>0.05^b^
Surgery	15(83.3)	34(87.2)	1.0^b^
Biopsy	3(16.7)	5(12.8)	

^a^ Calculated with a t-test

^b^ Calculated with a Fisher’s exact test or an χ^2^ test

### Comparison of CT features between hypo-PNETs and PDAC

#### Qualitative analysis

**Fig 2:**
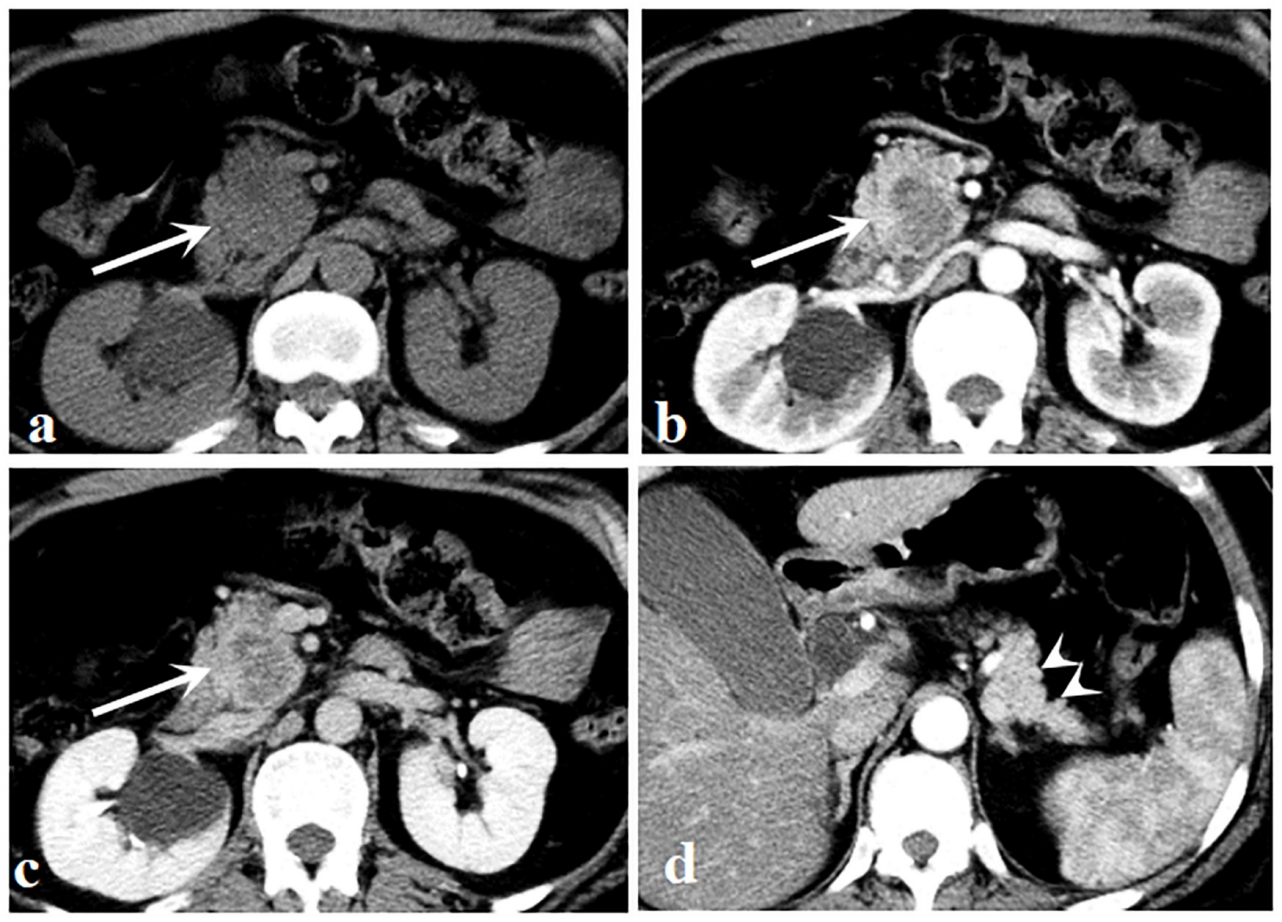
A 51-year-old woman with pancreatic neuroendocrine carcinoma. (a) Unenhanced image shows an isodense mass (arrow) located in the head of the pancreas. (b, c) Arterial (b) and portal venous (c) phase images show a well circumscribed mass (arrows) with a hypovascular enhancement pattern. (d) There was neither upstream pancreatic parenchymal atrophy nor pancreatic duct dilatation (arrowheads).

**Fig 3:**
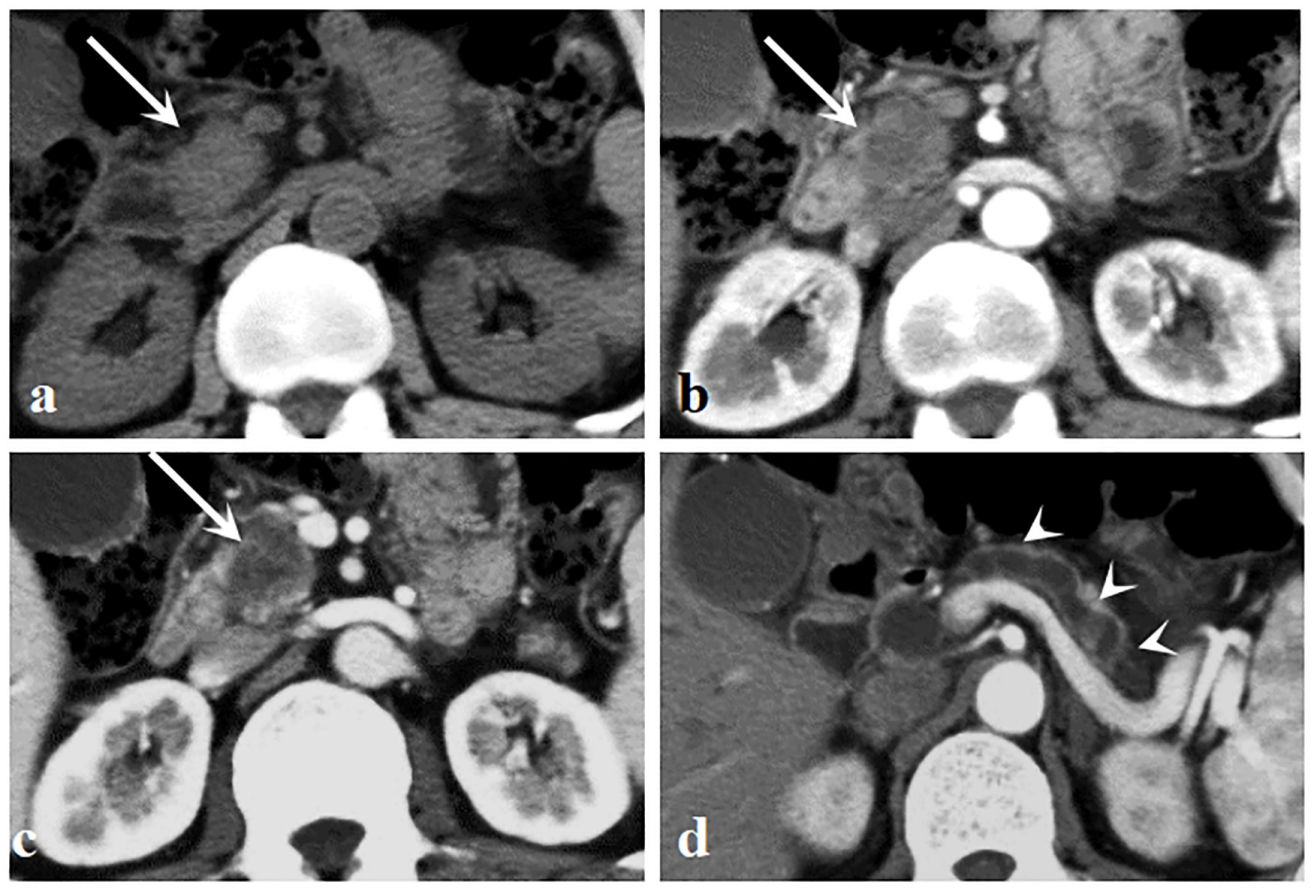
A 60-year-old man with pancreatic ductal adenocarcinoma. (a) Unenhanced image shows an isoattenuation mass (arrow) located in the head of the pancreas. (b, c) Arterial (b) and portal venous (c) phase images show an ill-defined mass (arrows) with a hypovascular enhancement pattern. (d) Pancreatic parenchymal atrophy and pancreatic duct dilatation were observed (arrowheads).

**Fig 4:**
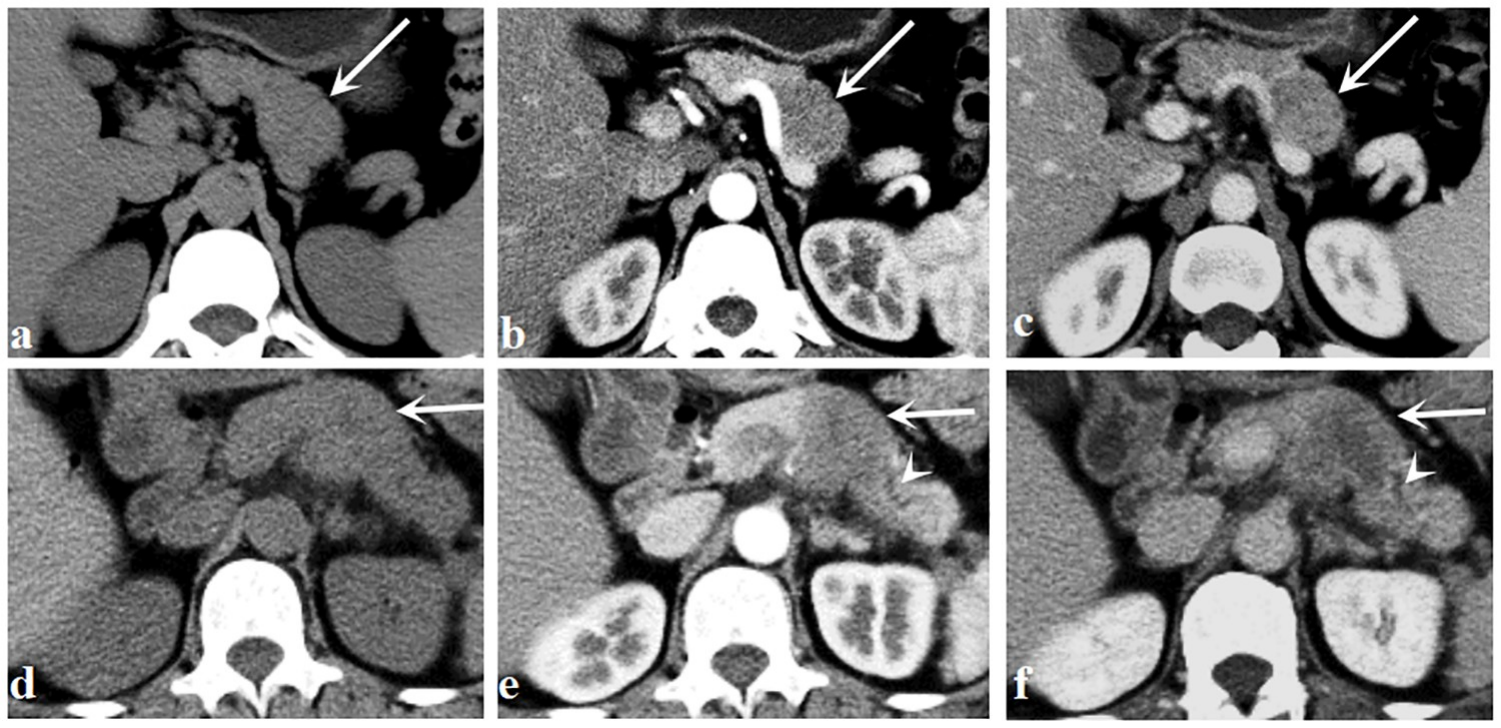
A 42-year-old man with pancreatic neuroendocrine carcinoma. (a—c) Unenhanced (a), arterial (b), and portal venous phase (c) images show a well-defined mass (arrows) in the body of the pancreas. The CT attenuation was 38 HU at the unenhanced phase, 52 HU at the arterial phase, and 75 HU at the portal venous phase. A 60-year-old man with pancreatic ductal adenocarcinoma. (d—f) Unenhanced (d), arterial (e), and portal venous phase (f) images show an ill-defined mass (arrows) in the body of the pancreas. The CT attenuation was 38 HU at the unenhanced phase, 43 HU at the arterial phase, and 55 HU at the portal venous phase. The dialated pancreatic duct is noted (arrowheads).

Considering all evaluated CT features, there were 2 patients evaluated by discussion between 2 radiologists and 3 patients evaluated by the third radiologist. Qualitative CT findings between hypo-PNETs and PDAC are summarized in Table 2. Tumors were located as follows in hypo-PNETs: 9 in the head of the pancreas, 3 in the body, and 6 in the tail. Tumors were located as follows in PDAC: 22 in the head of the pancreas, 11 in the body, and 6 in the tail. A well-defined margin was more common in hypo-PNETs compared with PDAC (*p* = 0.016). Pancreatic duct dilatation and local invasion or metastases were significantly more frequent in PDAC than those of hypo-PNETs. Pancreatic duct dilatation was found in 59% (23/39) of PDAC and local invasion or metastases was found in 79.5% (31/39) of PDAC, while only 11.1% (2/18) and 38.9% (7/18), respectively, of hypo-PNETs exhibited such. No significant differences were found in calcification, bile duct dilatation, or pancreatic atrophy between those two tumors. Representative cases of hypo-PNETs and PDAC are shown in Figs 2 and 3. CT attenuation of hypo-PNETs was higher than that of PDAC at the arterial and portal venous phase, as is shown in Fig 4.

**Table 2:** CT findings in hypovascular pancreatic neuroendocrine tumors (hypo-PNETs) and pancreatic ductal adenocarcinoma (PDAC).

CT findings	hypo-PNETs (n = 18)	PDAC (n = 39)	*P* value	Correlation coefficient
Location			0.278^b^	0.141
Head	9 (50)	22 (56.4)		
Body	3 (16.7)	11 (28.2)		
Tail	6 (33.3)	6 (15.4)		
Margin			0.016^b^	0.320
Well-defined	10 (55.6)	9 (23.1)		
Ill-defined	8 (44.4)	30 (76.9)		
Calcification	1 (5.6)	3 (7.7)	1.0^b^	0.039
Pancreatic duct dilatation	2(11.1)	23 (59)	0.002^b^	0.448
Bile duct dilatation	2 (11.1)	14 (35.9)	0.106^b^	0.256
Pancreatic Atrophy	4 (22.2)	20 (51.3)	0.076^b^	0.274
Local invasion or Metastases	7 (38.9)	31 (79.5)	0.003^b^	0.400
Size (cm)	4.3±1.6	3.8±1.3	0.20^a^	0.242
Tumor contrast enhancement (HU)				
Arterial phase	59.8±8.7	44.2±9.2	<0.001^a^	0.630
Portal venous phase	69.0±8.9	60.0±10.4	0.003^a^	0.393
Delayed phase	66.6±10.6	60.6±10.5	0.083^a^	0.261
Tumor-to-pancreas enhancement ratio				
Arterial phase	0.66±0.14	0.51±0.10	<0.001^a^	0.536
Portal venous phase	0.71±0.15	0.61±0.12	0.010^a^	0.338
Delayed phase	0.79±0.11	0.73±0.14	0.174^a^	0.206

^a^ Calculated with a t-test

^b^ Calculated with a Fisher’s exact test or an χ^2^ test

#### Quantitative analysis

**Fig 5:**
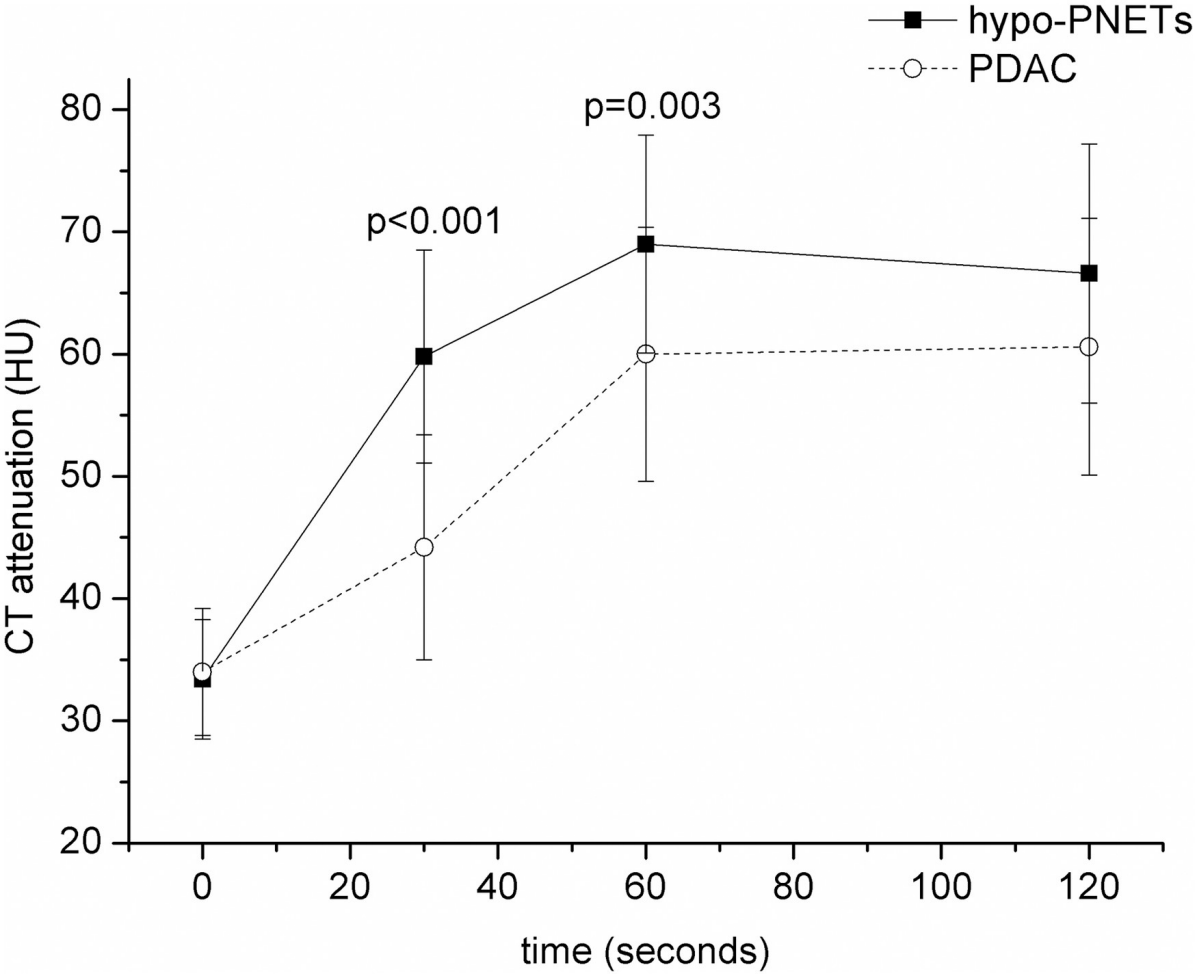
The dynamic contrast-enhanced curves in hypo-PNETs and PDAC. The CT attenuation of hypo-PNETs and PDAC was 33.4 ± 4.9 and 34.0 ± 5.2 Hounsfield units at the unenhanced CT images, respectively. The CT attenuation of hypo-PNETs was higher than that of PDAC in the arterial phase (p<0.001) and portal venous phases (p = 0.003). hypo-PNET, hypovascular pancreatic neuroendocrine tumor; PDAC, pancreatic ductal adenocarcinoma.

**Fig 6:**
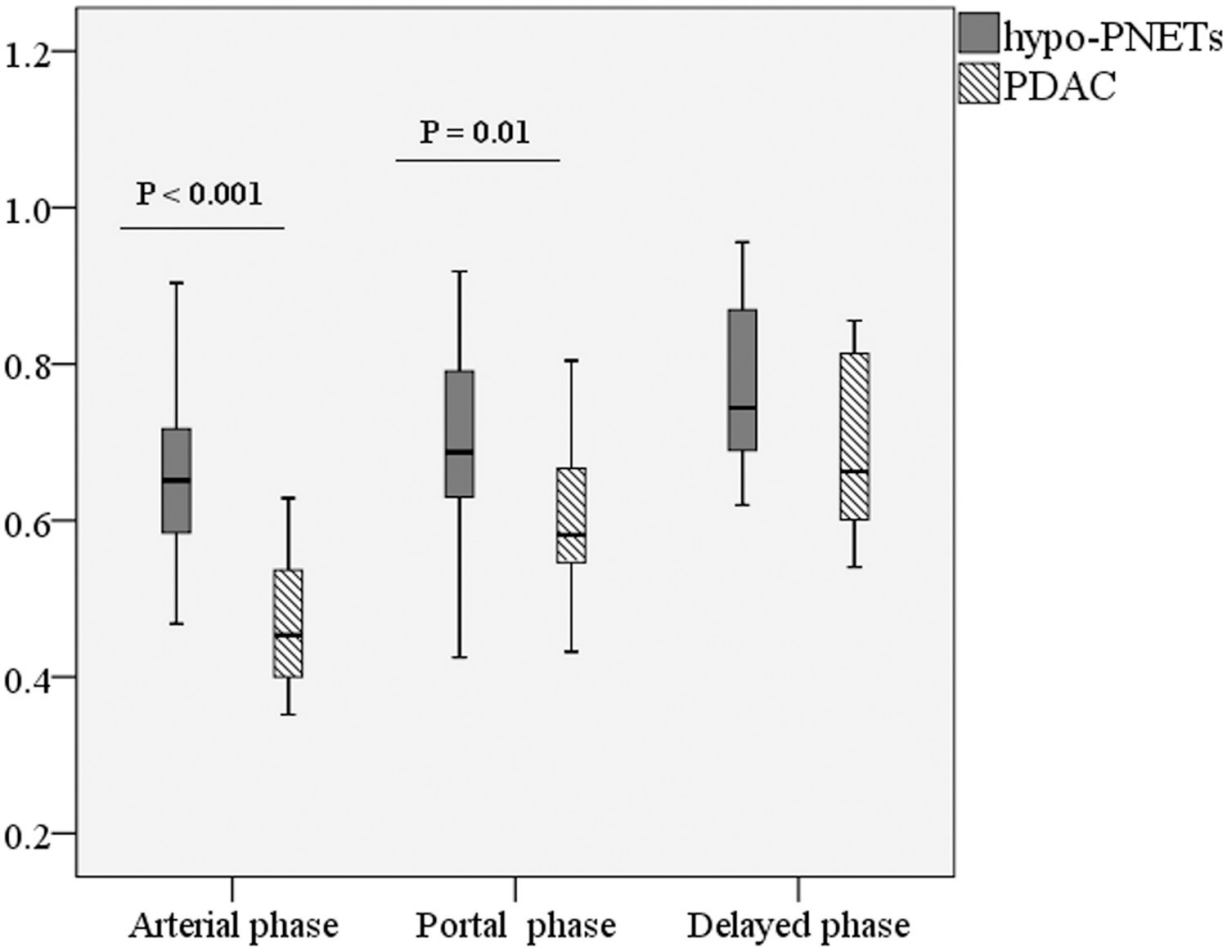
Box-and-whisker plots of the tumor-to-pancreas enhancement ratio in hypo-PNETs and PDAC in the arterial, portal venous, and delayed phase. The tumor-to-pancreas enhancement ratios in hypo-PNETs were higher than those of PDAC in the arterial phase (p<0.001) and portal venous phase (p = 0.01). hypo-PNET, hypovascular pancreatic neuroendocrine tumor; PDAC, pancreatic ductal adenocarcinoma.

Quantitative CT findings between hypo-PNETs and PDAC are summarized in Table 2. There was no significant difference in tumor size between the two groups (4.3cm ± 1.6 vs 3.8cm ± 1.3, p = 0.242). The attenuation of hypo-PNETs at the arterial phase (59.8 HU ± 8.7 vs 44.2 HU ± 9.2) and portal venous phase (69.0 HU ± 8.9 vs 60.0 HU ± 10.4) was significantly higher than that of PDAC (*p* < 0.001, *p* = 0.003, respectively), while there was no significant difference in delayed CT attenuation between hypo-PNETs and PDAC (66.6 HU ± 10.6 vs 60.6 HU ± 10.5, p = 0.261) (Fig 5). The tumor-to-pancreas enhancement ratios of hypo-PNETs at the arterial phase (0.66 ± 0.14 vs 0.51 ± 0.10) and portal venous phase (0.71 ± 0.15 vs 0.61 ± 0.12) were significantly higher than those of PDAC (*p* < 0.001, *p* = 0.01, respectively), while there was no significant difference in delayed tumor-to-pancreas enhancement ratio between the two groups (0.79 ± 0.11 vs 0.73 ± 0.14, p = 0.174) (Fig 6).

### Diagnostic performance of CT findings in differentiating hypo-PNETs from PDAC

**Fig 7:**
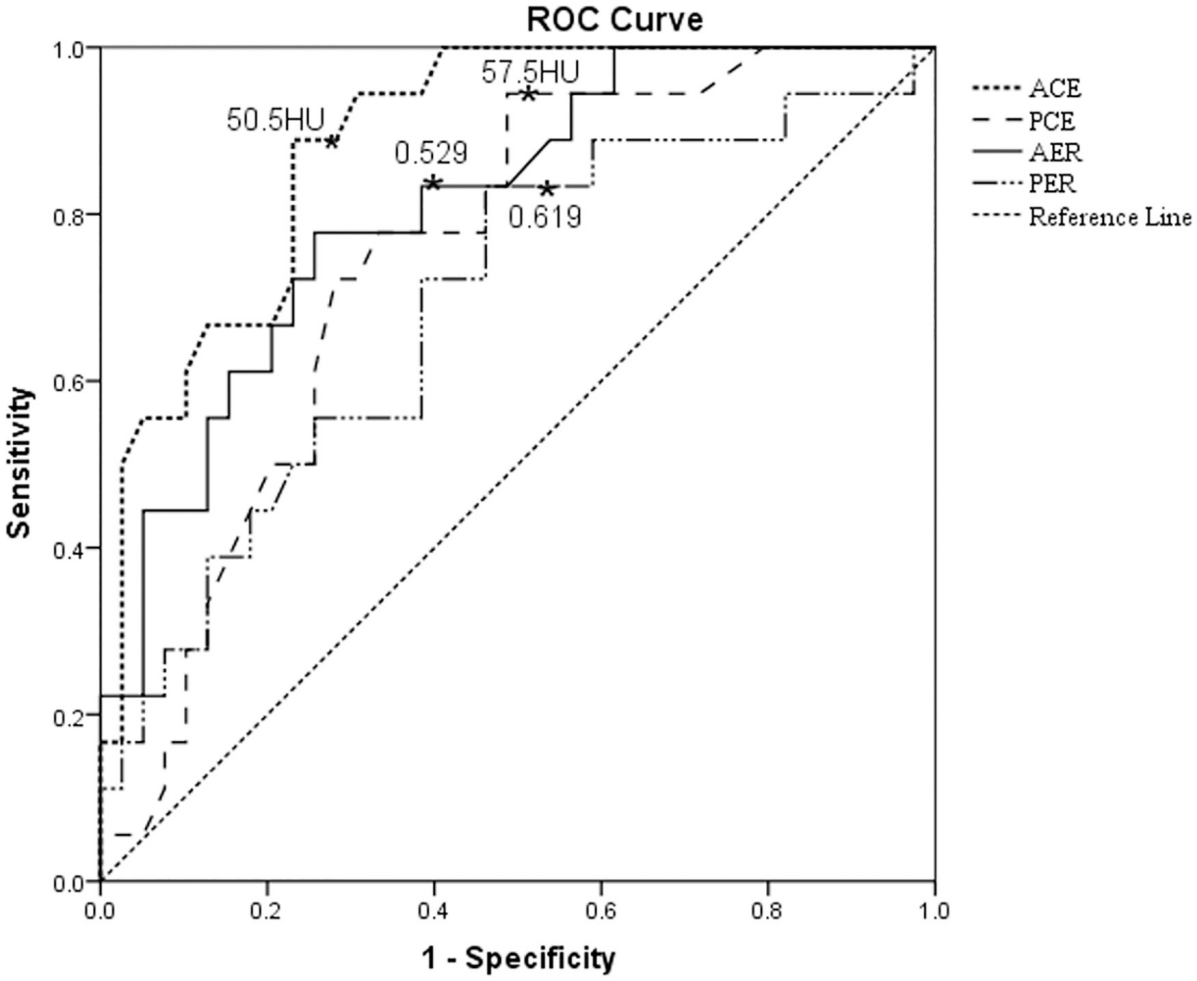
Receiver operating characteristic curves of quantitative computed tomography imaging findings in the differentiation of hypo-PNETs from PDAC. The area under the curves ranged from 0.693–0.888. hypo-PNET, hypovascular pancreatic neuroendocrine tumor; PDAC, pancreatic ductal adenocarcinoma; ACE, contrast enhancement in the arterial phases; PCE, contrast enhancement in the portal venous phases; AER, the tumor-to-pancreas enhancement ratio in the arterial phase; PER, the tumor-to-pancreas enhancement ratio in the portal venous phases.

The area under the curve (AUC), sensitivity, specificity, odds ratio, and 95% confidence intervals for each CT finding that could be used to differentiate hypo-PNETs from PDAC are summarized in Table 3. The sensitivity and specificity of the imaging features for hypo-PNETs identification (vs. PDAC) ranged from 44.4% - 94.4% and 53.9% - 88.9%, respectively. The AUC ranged from 0.607–0.888. The CT attenuation and tumor-to-pancreas enhancement ratio at the arterial phase showed the largest AUC (0.888 and 0.812) with 83.3–88.9% of sensitivity and 61.6–77.0% of specificity (Fig 7). Pancreatic duct dilatation, local invasion or metastasis, and CT attenuation and tumor-to-pancreas enhancement ratio at the portal venous phase also showed acceptable AUC (0.703–0.748).

**Table 3:** The receiver operating characteristic analysis of CT findings in differentiating hypovascular pancreatic neuroendocrine tumors (hypo-PNETs) from pancreatic ductal adenocarcinoma (PDAC).

CT findings	AUC	Sensitivity (%)	Specificity (%)	Odds ratio	Confidence interval (95%)
Tumor margin	0.607	44.4	76.9	0.375	0.114–1.234
Pancreatic duct dilatation	0.739	59	88.9	11.5	2.316–57.101
Local invasion or Metastases	0.703	79.5	61.1	6.089	1.788–20.741
Tumor contrast enhancement (arterial)	0.888	88.9	77	0.839	0.765–0.921
Tumor contrast enhancement (portal)	0.748	94.4	54.3	0.917	0.863–0.975
Tumor-to-pancreas enhancement ratio (arterial)	0.812	83.3	61.6	0.034	0–4.444
Tumor-to-pancreas enhancement ratio (portal)	0.693	83.3	53.9	0.003	0–0.357

The optimal cut-off value of CT attenuation by maximizing the sum of sensitivity and specificity was 50.5 HU at the arterial phase with 88.9% of sensitivity and 77% of specificity, and 57.5 HU at the portal venous phase with 94.4% of sensitivity and 54.3% of specificity (Fig 7). The optimal cut-off value of tumor-to-pancreas enhancement ratio was 0.529 at the arterial phase with 83.3% of sensitivity and 61.6% of specificity, and 0.619 at the portal venous phase with 83.3% of sensitivity and 53.9% of specificity (Fig 7).

## 4. Discussion

PNETs usually demonstrated a hypervascular pattern on CT or MRI imaging. However, hypovascualr PNETs are also reported in several studies [11, 12]. Since there are overlaps in imaging findings between hypo-PNETs and PDAC, hypo-PNETs are often masqueraded as PDAC [18, 19]. CT is an important imaging modality in the diagnosis and staging of PNETs [20, 21]. Although CT can’t replace pathology assessment of tumor confirmation, knowing the type of tumor may be helpful and beneficial in estimating the aggressiveness of the tumor and deciding a treatment plan before surgery. In the present study, we showed that CE-CT is helpful in the differentiation between hypo-PNETs and PDAC. Tumor margin, pancreatic duct dilatation, local invasion or metastasis, and CT contrast enhancement and tumor-to-pancreas enhancement ratio in the arterial phase and portal venous phase are useful predictors for the differentiation of those two tumors.

Previous studies reported that common CT findings of PNETs included a hypervascular enhancement pattern with a clear margin, no ductal dilatation and lack of vascular invasion [10, 22]. Our study evaluated the CT features of hypo-PNETs and compared with PDAC. Tummala et al. [23] evaluated the incidence of pancreatic duct dilatation in malignant tumors. They demonstrated that 152 of 187 patients with pancreatic duct dilatation had malignant tumors. Among these 152 patients, 134 (88%) were confirmed with PDAC and 14 (9%) were confirmed with PNETs. Consequently, pancreatic duct dilatation is considered to be an important indicator for PDAC identification (vs PNETs). In our study, pancreatic duct dilatation was more frequent in PDAC compared with hypo-PNETs (59% vs 11.1%, p = 0.002), which was consistent with previous study. This differentiation of pancreatic duct dilatation can be explained that PNETs originate from progenitor islet cells in the pancreas parenchyma, while PDAC originates from the ductal epithelium. In addition, we also found a statistical significance between those two tumors with respect to tumor margin and local invasion or metastasis, which is analogous with results of Guo et al. [14] in that both *p* <0.01 for those two CT findings. Thirty-seven patients with PDAC and thirteen patients with hypovascular PNEC were finally included in their study. CT features, including tumor margin, local invasion or metastasis, signal intensity at all three phases and signal intensity ratios at the arterial and portal venous phase, and ADC values, showed statistical significance between the two groups. We also investigated the diagnostic performance of pancreatic duct dilatation, tumor margin, and local invasion or metastasis in differentiation of hypo-PNETs from PDAC. We found that pancreatic duct dilatation had the largest specificity of 88.9%, although the sensitivity was lower than that of local invasion or metastasis (59.5% vs. 75%).

PNETs are a heterogeneous group with varying clinical presentations [17]. In our study, 18 of 84 (21.4%) PNETs showed a hypovascular pattern at the arterial phase, which is in agreement with previous study [19], where 22% of PNETs showed hypovascularity at the arterial phase. Guo et al. [24] investigated the values of CT imaging features in differentiation of PDAC from PNEC, 28 patients with PDAC and 14 patients with PNEC were finally included into the study. They found that CT attenuation values and contrast ratios of PNEC at the arterial and portal venous phase were higher than those of PDAC. Our results also showed that CT attenuation values at the arterial and portal venous phase can be one of deciding factors in differentiation of hypo-PNETs from PDAC, which was consistent with previous study [24]. An arterial threshold of 50.5 HU and a portal venous threshold of 57.5 HU can be used to differentiate hypo-PNETs from PDAC with high sensitivities of 88.9% and 94.4%, and high specificities of 77% and 54.3%, respectively.

Kim et al. [12] investigated the portal venous enhancement ratio for differentiating grade 3 from grade 1/2 pancreatic neuroendocrine neoplasms. Belousova et al. [15] investigated the diagnostic accuracy of the arterial enhancement ratio in determining the grade 2 PNETs. In our study, we evaluated the diagnostic performance of the arterial and portal venous tumor-to-pancreas enhancement ratio in differentiating hypo-PNETs from PDAC. Tumor-to-pancreas enhancement ratio of 0.529 at the arterial phase and 0.619 at the portal venous phase may be used as thresholds to differentiate hypo-PNETs from PDAC. However, the sensitivities and specificities of tumor-to-pancreas enhancement ratios were lower than those of tumor attenuation at the arterial and portal venous phase.

Our study had several possible limitations. First, various types of CT scanners were used in this study. These limitations could not be avoided as this study itself was retrospective. Second, we could not obtain inter-observer variability of the qualitative image analysis due to the consensus review by radiologists. However, the discrepancy was minor during imaging analysis between two radiologists. Third, we used a fixed-delay method for contrast-enhanced CT, and the enhanced images were collected at 30s for the arterial phase, not the so-called pancreatic parenchymal phase. But considering the objective of our study is to provide information for prompt decision making, results of our study could be applicable in clinical practice if further studies followed. Fourth, only CE-CT was investigated. The role of other imaging modalities, particularly endoscopic ultrasound-guided fine-needle aspiration (EUS-FNA) or hybrid techniques (e.g. octreotide SPECT) may be helpful in diagnosis of those two tumors [25–27]. Further studies addressing the diagnostic reliability of other imaging modalities or comparisons between CT and EUS-FNA or octreotide SPECT are needed. Moreover, further studies should be followed to include more hypo-PNET or PDAC patients that have not been included in our present study to validate our diagnostic criteria and make the study more satisfactory. Our study is consequential for the suggestion of possibility to distinguish between hypo-PNETs and PDAC through qualitative and quantitative CT features, such as tumor attenuation and tumor-to-pancreas enhancement ratio at the arterial phase.

## 5. Conclusions

In conclusion, PDAC usually showed an ill-defined margin, pancreatic duct dilatation, and local invasion or metastasis compared with hypo-PNETs. Moreover, our data indicated that quantitative CT parameters, especially tumor contrast enhancement and enhancement ratio at the arterial phase, played a more important role than qualitative analysis in differentiating those two tumors.
